# Supplementary material for: Predictive maps in rats and humans for spatial navigation
Source: Curr Biol. 2022 Sep 12;32(17):3676–3689.e5. doi: 10.1016/j.cub.2022.06.090 (PMC9616735; doi:10.1016/j.cub.2022.06.090)
Supplement: Document S1. Figures S1–S5 and Tables S1 and S2 [file mmc1.pdf]

Current Biology, Volume *32*

Supplemental Information

Predictive maps in rats and humans

for spatial navigation

William de Cothi, Nils Nyberg, Eva-Maria Griesbauer, Carole Ghanamé, Fiona Zisch, Julie M. Lefort, Lydia Fletcher, Coco Newton, Sophie Renaudineau, Daniel Bendor, Roddy Grieves, Éléonore Duvelle, Caswell Barry, and Hugo J. Spiers

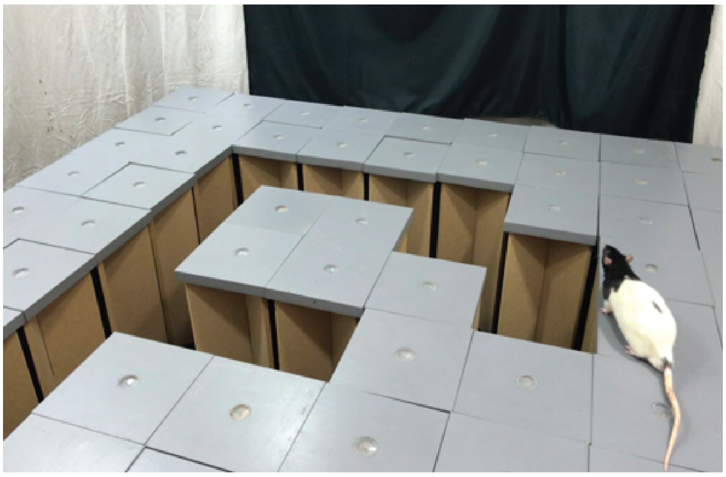

Figure S1: The maze environment used for the rat experiment, related to Figure 1. The environment consists of 100 removable mazes modules with a black curtain over one of the surrounding edges to provide a single extra-maze cue. Reward can be dispensed at the goal module by filling the well with chocolate milk via polymeric tubing beneath the maze.

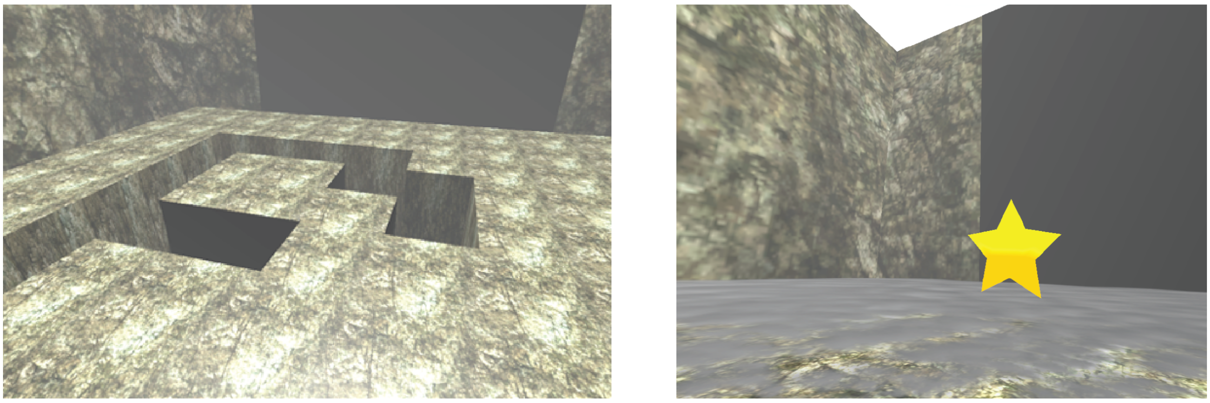

Figure S2: The virtual environment used for the human experiment, related to Figure 1. The environment had the same proportions as the rat environment and consisted of 100 removable mazes modules with a black curtain over one of the surrounding edges to provide a single extra-maze cue. A seamless texture was applied to the maze modules and walls and a fog lined the floor of the maze (see right image) to ensure humans had to rely on spatial memory to understand the maze structure. Reward was indicated by a gold star that would appear at the goal module when the participant successfully navigated to it.

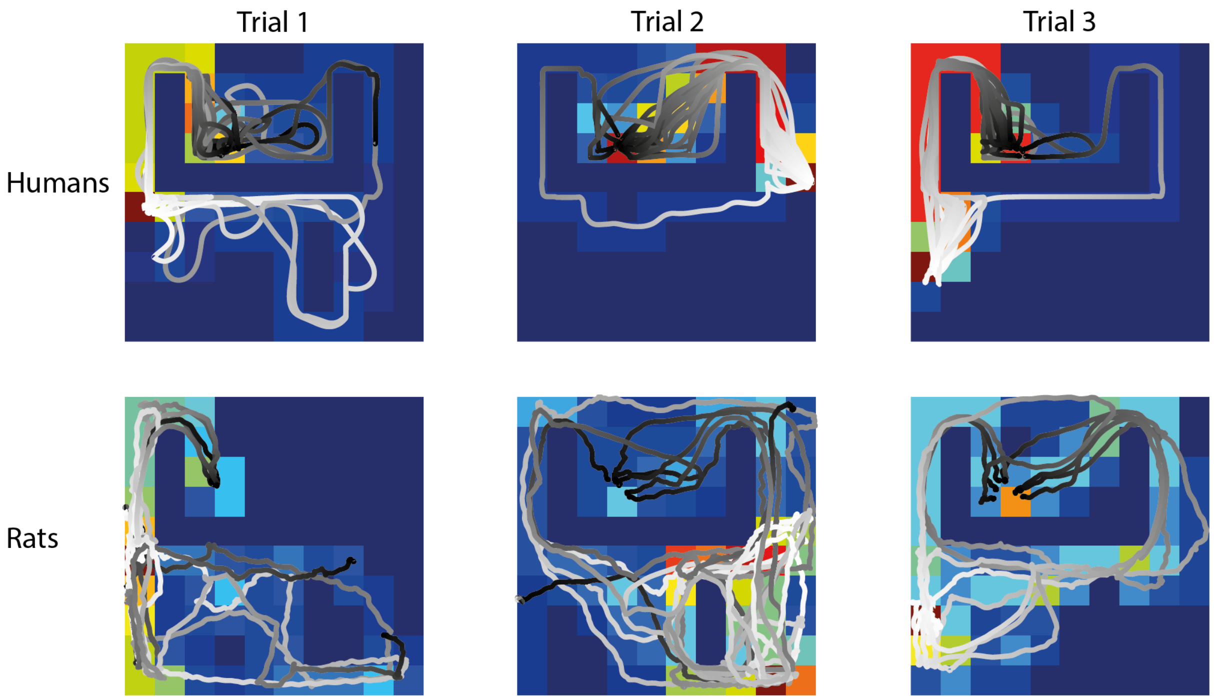

Figure S3: The route choices of humans and rats were often suboptimal at the start of a new maze configuration, related to Figure 2. Examples of the human (top) and rat (bottom) trajectories overlaying occupancy maps for the first 3 trials on maze configuration 20. The white-black colour gradient shows the beginning-end of each trajectory. Initially the paths taken by the humans and rats were often suboptimal (leftmost column) with performance generally improving rapidly within the first 3 trials of a new maze configuration. The goal location is 4 squares right and down from the top-left corner.

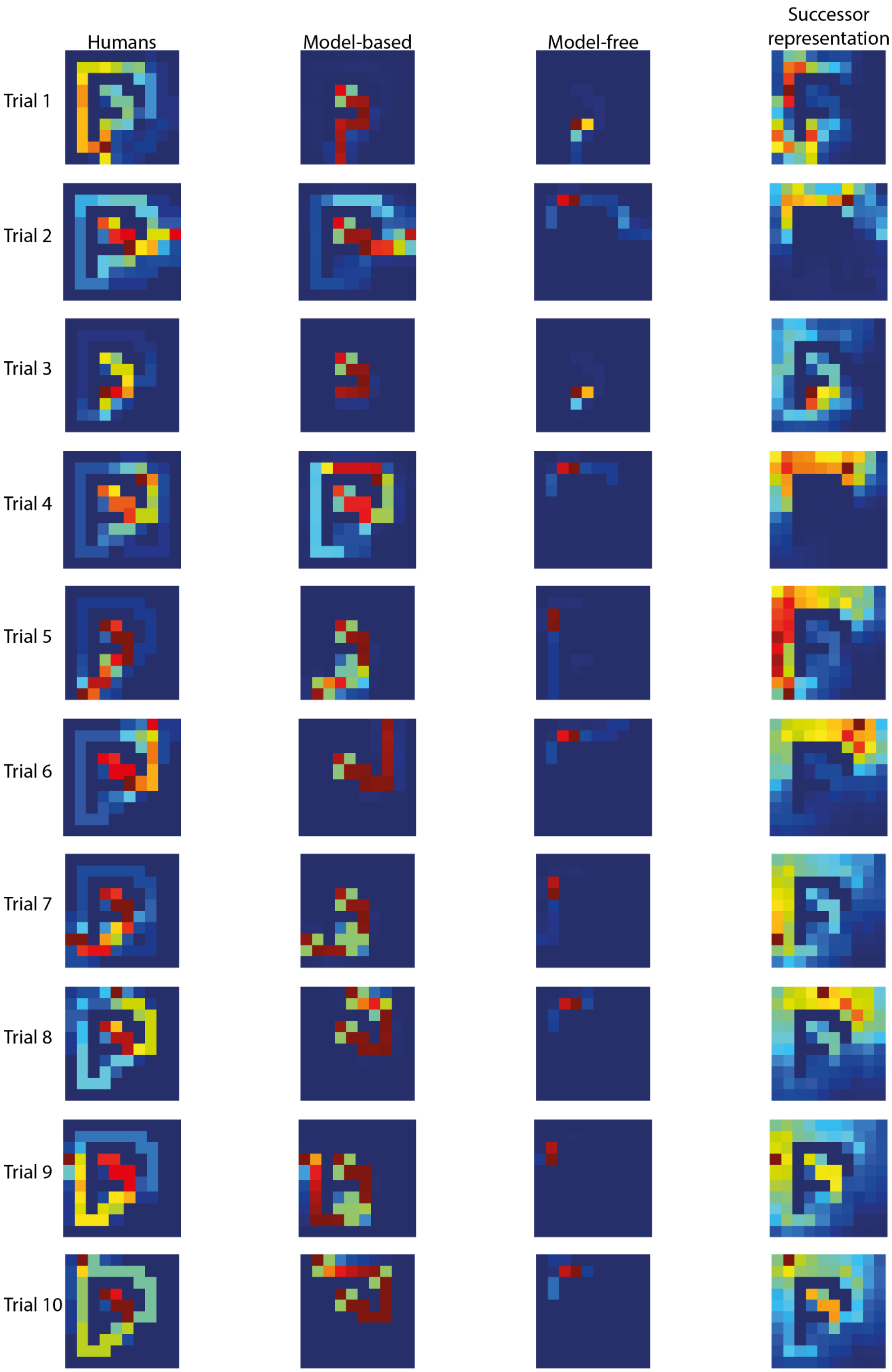
Humans
Model-based
Model-free
Successor representation
Trial 1
Trial 2
Trial 3
Trial 4
Trial 5
Trial 6
Trial 7
Trial 8
Trial 9
Trial 10

Figure S4: Human and agent occupancy maps for maze configuration 21, related to Figure 6. The occupancy maps of the humans (leftmost column) and agents for each of the 10 trials (rows) on maze configuration 21. The Model-Based agent (second column) quickly learns an accurate model of the environment and uses it to choose the shortest route to the goal with respect to that model (goal location is 4 squares right and down from the top-left corner). Conversely, the model-free agent (third column) is unable to update its value representation fast enough to successfully adapt to the new maze configuration, and particularly struggles on later trials where the starting position requires longer and more tortuous routes. The successor representation agent (rightmost column) sits on the spectrum between model-based and model-free methods, initially struggling to find an efficient route to the goal but providing a good match to the human behaviour on later trials.

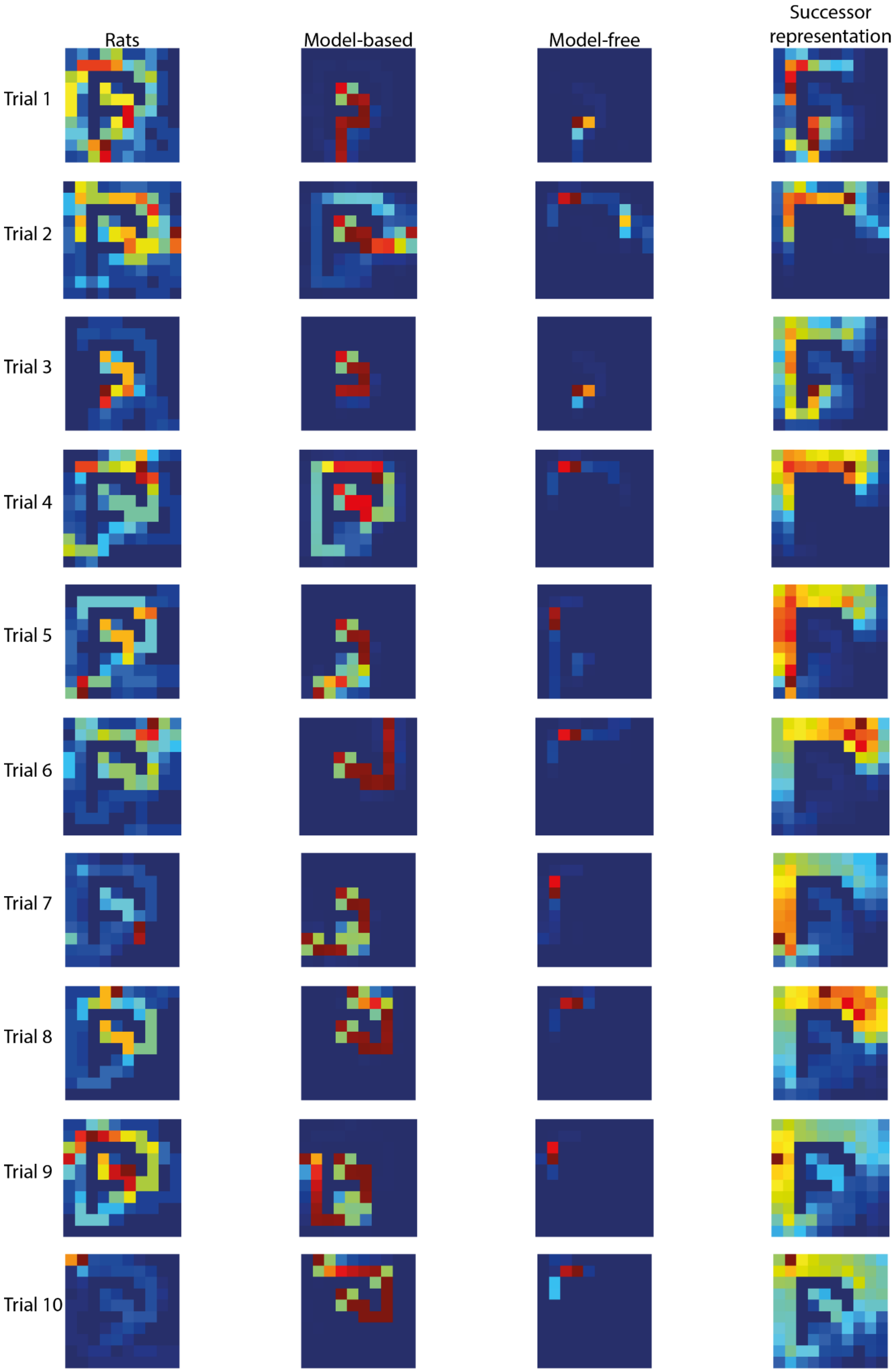
Rats
Model-based
Model-free
Successor representation
Trial 1
Trial 2
Trial 3
Trial 4
Trial 5
Trial 6
Trial 7
Trial 8
Trial 9
Trial 10

Figure S5: Rat and agent occupancy maps for maze configuration 21, related to Figure 6. The occupancy maps of the rats (leftmost column) and agents for each of the 10 trials (rows) on maze configuration 21. The Model-Based agent (second column) quickly learns an accurate model of the environment and uses it to choose the shortest route to the goal with respect to that model (goal location is 4 squares right and down from the top-left corner). Conversely, the model-free agent (third column) is unable to update its value representation fast enough to successfully adapt to the new maze configuration, and particularly struggles on later trials where the starting position requires longer and more tortuous routes. The successor representation agent (rightmost column) sits on the spectrum between model-based and model-free methods, initially struggling to find an efficient route to the goal but providing a good match to the rat behaviour on later trials.

	α			γ		
Agent	MF	MB	SR	MF	MB	SR
Participant 1	0.09	1	1	0.43	0.80	0.76
Participant 2	0.08	1	0.89	0.43	0.80	0.79
Participant 3	0.11	1	1	0.51	0.81	0.82
Participant 4	0.30	1	0.96	0.62	0.82	0.81
Participant 5	0.07	1	0.80	0.41	0.80	0.77
Participant 6	0.11	1	0.81	0.60	0.82	0.85
Participant 7	0.07	1	1	0.40	0.79	0.78
Participant 8	0.15	1	1	0.54	0.80	0.77
Participant 9	0.09	1	0.93	0.50	0.80	0.77
Participant 10	0.19	1	0.82	0.64	0.83	0.84
Participant 11	0.11	1	0.92	0.54	0.81	0.78
Participant 12	0.16	1	0.98	0.55	0.80	0.75
Participant 13	0.12	1	1	0.52	0.80	0.77
Participant 14	0.12	1	0.94	0.51	0.80	0.76
Participant 15	0.14	1	0.78	0.56	0.80	0.75
Participant 16	0.13	1	1	0.59	0.81	0.80
Participant 17	0.13	1	0.77	0.62	0.82	0.85
Participant 18	0.19	1	0.91	0.55	0.80	0.75

Table S1: Human behaviour maximum likelihood parameters, related to Figure 3. The learning rates α and discount factors γ for the model-free (MF), model-based (MB) and successor representation (SR) agents, calculated for each individual.

	α			γ		
Agent	MF	MB	SR	MF	MB	SR
Rat 1	0.07	1	0.76	0.43	0.79	0.80
Rat 2	0.11	1	0.77	0.48	0.80	0.83
Rat 3	0.12	1	0.87	0.45	0.79	0.71
Rat 4	0.65	1	0.87	0.01	0.78	0.76
Rat 5	0.42	1	0.77	0.01	0.78	0.77
Rat 6	0.09	1	0.90	0.34	0.79	0.79
Rat 7	0.12	1	0.70	0.16	0.80	0.82
Rat 8	0.08	1	0.91	0.28	0.78	0.81
Rat 9	0.07	1	0.87	0.42	0.80	0.81

Table S2: Rat behaviour maximum likelihood parameters, related to Figure 3. The learning rates α and discount factors γ for the model-free (MF), model-based (MB) and successor representation (SR) agents, calculated for each individual.
